# Cluster-Like Headache Secondary to Anamnesis of Sphenoid Ridge Meningioma: A Case Report and Literature Review

**DOI:** 10.3389/fneur.2019.00023

**Published:** 2019-01-25

**Authors:** Liang Kou, Jinsha Huang, Yan Xu, Chao Han, Kai Ma, Xingfang Guo, Yun Xia, Fang Wan, Sijia Yin, Junjie Hu, Jiawei Wu, Yadi Sun, Guoxin Zhang, Ling Liu, Nian Xiong, Tao Wang

**Affiliations:** ^1^Department of Neurology, Union Hospital, Tongji Medical College, Huazhong University of Science and Technology, Wuhan, China; ^2^Department of Neurology, The First Affiliated Hospital of USTC, Division of Life Sciences and Medicine, University of Science and Technology of China, Hefei, China

**Keywords:** cluster headache, craniotomy, glial hyperplasia, meningioma, postoperative headache, symptomatic

## Abstract

Cluster headache is generally considered to be a primary headache; secondary cluster-like headache is quite rare, while cluster-like headache secondary to meningioma is even rarer. Here, we describe an unusual case with cluster-like headache 2.5 years after sphenoid ridge meningioma surgery. The cluster-like headache and meningioma were on the same side, and even at the same position. Furthermore, the cluster-like headache lasted for 6 months. In addition, the patient did not respond well to conventional treatments for cluster headache, such as oxygen inhalation, carbamazepine, and tramadol. Brain magnetic resonance imaging demonstrated a softening lesion, glial hyperplasia, and localized thickening and enhancement of the dura in the left frontal-temporal lobe. However, positron-emission computed tomography showed reduced metabolism in the left frontal-temporal lobe. Although the possibility of a primary headache cannot be completely eliminated, the association between cluster-like headache and probable tumor recurrence or postoperative changes should be considered.

## Introduction

Cluster headache is a typical severe unilateral episode of headache, usually lasting 15–180 min, with a frequency of once every other day to eight times a day, associated with autonomic symptoms ipsilateral to the pain, or a feeling of restlessness, or both (1). According to epidemiological surveys in the United States and Europe, the prevalence of cluster headache in the general population is about 0.1%, however, it is unclear whether the prevalence varies geographically (2). Cluster headache is more common in males, and the mean age of onset is about 30 years (3). The disease duration has not been well studied, but it can exceed 15 years in most patients. Due to the high frequency of the attacks and long course of the disease, the syndrome has a great impact on the quality of life of patients, increasing the burden on individuals and the society (4).

In the present study, we describe the case of a 40-year-old man who experienced an onset of cluster-like headache after the surgical removal of sphenoid ridge meningioma 2.5 years prior. We also provide a review of the literature on cluster-like cases secondary to meningioma and pterional craniotomy and discuss the possible pathophysiological mechanisms.

## Case Report

**Figure 1:**
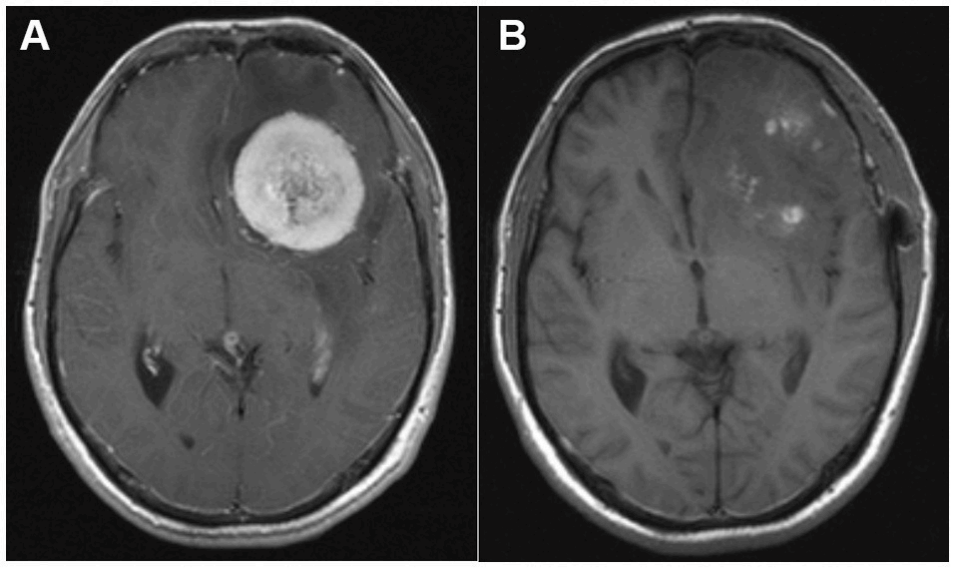
**(A)** Pre-operative magnetic resonance imaging (MRI; T1-weighted sequence after gadolinium enhancement) showing a large mass with regular borders and surrounding edema, exerting a mass effect on the adjacent frontal lobe and a 12-mm left-to-right midline shift. **(B)** Post-operative MRI (T1-weighted sequence after gadolinium enhancement) demonstrating complete tumor removal.

A 37-year-old man with 15-day history of headache was admitted to the Department of Neurosurgery of Wuhan Union Hospital in June 2015. The headache was characterized as a distending pain associated with nausea. Despite a family history of migraine, the patient had not experienced a headache previously. He did not smoke or drink alcohol. His general physical and neurological examination results were normal. Routine laboratory test results, electrocardiogram, and chest X-ray were unremarkable. Brain magnetic resonance imaging (MRI) with gadolinium enhancement showed a large mass with regular borders and surrounding edema, exerting a mass effect on the adjacent frontal lobe (Figure 1A). Surgery was suggested and successfully performed, with complete resection, as indicated in the postoperative MRI (Figure 1B). Surgical biopsy confirmed the diagnosis of a transitional meningioma, WHO grade I. After the operation, the patient's headache was completely relieved.

He remained free of all forms of headaches until November 2017, when he developed a strictly left-side periorbital pain in the absence any specific triggering factor. The attacks were accompanied by ipsilateral rhinorrhea, lacrimation, eyelid edema, ptosis, and bilateral photophobia. The attacks lasted 3–4 h and occurred daily, in the afternoon. During the attacks, the patient was restless and between the attacks, the patient was pain-free. He was treated in the first instance with carbamazepine, however, yielding poor efficacy; tramadol only provided partial relief.

**Figure 2:**
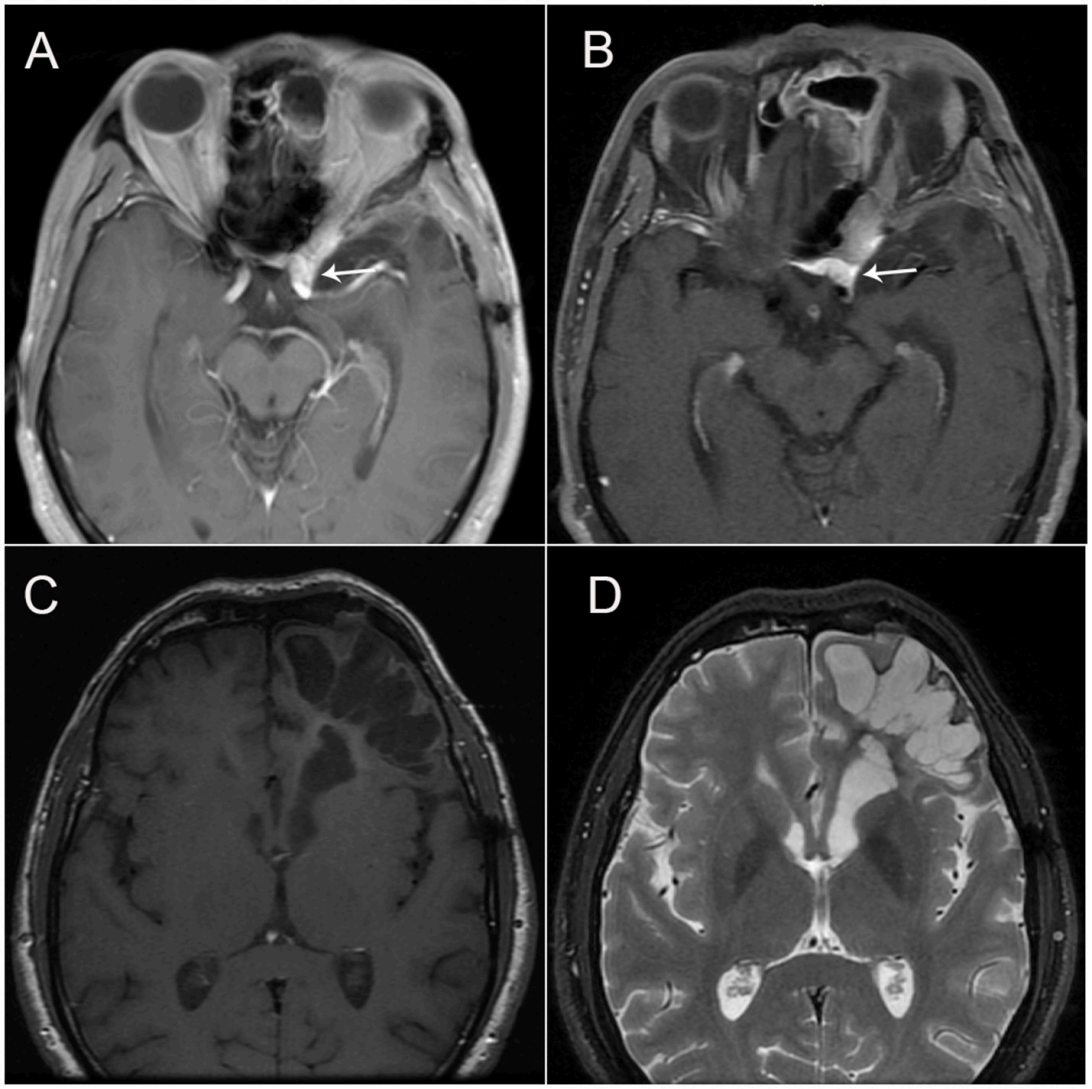
**(A)** Brain magnetic resonance imaging (MRI; T1-weighted sequence after gadolinium enhancement) showing the fusiform thickening of the localized dura mater in the left frontal-temporal lobe, which is vividly enhanced with contrast; **(B)** Brain MRI (T1-weighted sequence) showing the fusiform thickening of the localized dura mater in the left frontal-temporal lobe. **(C,D)** Brain MRI (T1- and T2-weighted sequence) showing a large area of softening lesion and glial hyperplasia in the left frontal-temporal lobe.

**Figure 3:**
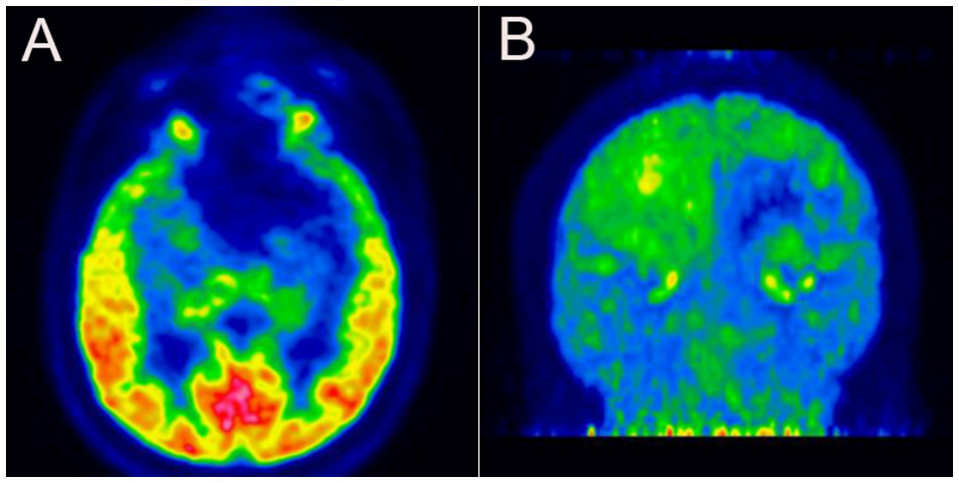
**(A,B)** ^18^F-fluorodeoxyglucose positron-emission tomography revealing low glucose metabolism in the left frontal-temporal lobe.

The patient visited our department 4 months after the onset of attacks. A few days prior to the visit (in December 2017), he had undergone a brain MRI, which showed a softening lesion, glial hyperplasia, and localized thickening and enhancement of the dura (Figure 2A) in the left frontal-temporal lobe. Considering the possibility of tumor recurrence, an MRI of the eyes and a whole-body PET scan were performed; however, the results were confusing. In March 2018, the MRI of the eyes demonstrated a stronger signal of the cerebral dura (Figure 2B), a large area of the softening lesion, and glial hyperplasia (Figures 2C,D) in the left frontal-temporal lobe, as observed previously. However, no signs of tumor were indicated on the ^18^F-FDG PET scan performed in February 2018 (Figures 3A,B). We consulted the Department of Neurosurgery and Radiology. The consensus was that, as the enhanced volume of the fronto-temporal lobe had not increased significantly, recurrence of the tumor could not be determined at that stage, and thus regular follow-up was recommended. Based on a comprehensive analysis of the patient's clinical manifestations and the International Classification of Headache Disorders (ICHD-3 beta) (1), we made a diagnosis of cluster-like headache.

During the attacks, oxygen therapy showed no effect. Treatment with methylprednisolone sodium succinate injection (40 mg per day) provided complete remission on the day after medication administration. The treatment was then shifted to oral administration of methylprednisolone tablets (40 mg per day), tapering to 8 mg per day. During this period, no cluster-like headache attacks occurred. However, the cortisone therapy did not result in permanent remission, as the cluster-like headache attacks recurred when methylprednisolone tablets were tapered to 4 mg per day. Thus, the dose of 8 mg per day was temporarily maintained. After 1 month of steroid therapy, acne appeared. Considering the side effects of steroids, the dosage was adjusted to one tablet every other day. During the 4 months of follow-up, there was no recurrence of headache.

## Discussion

We have reported an unusual case of a 40-year-old man who developed cluster-like headache 2.5 years after the surgical removal of sphenoid ridge meningioma. The cluster-like headache and meningioma occurred at the same position. Furthermore, the cluster-like headache had a 6-month duration. Typically, duration of episodic cluste headache is between 2 weeks and 3 months; thus, our patient showed an atypical duration. In addition, the patient did not respond well to conventional treatments for cluster headache, such as oxygen inhalation, carbamazepine, and tramadol. Therefore, although the possibility of primary cluster headache cannot be completely ruled out, this was more likely a case of secondary cluster-like headache.

Although usually considered as an idiopathic disorder, symptomatic cluster-like headaches have been previously reported; these can be secondary to neurovascular compression (5, 6), arteriovenous malformation (7), pituitary adenomas (8, 9), or other brain lesions (10, 11). Internal carotid artery dissection (12, 13), cataract surgery (14, 15), dental extraction (16), and barotrauma (17) have also been reported as a cause for symptomatic cluster-like headache.

Several cases of cluster-like headache secondary to meningioma have been reported, and all the patients had experienced complete relief after surgical resection (11, 18–21) (Table 1). The pathogenesis in these cases is not completely clear, however, may involve changes in the hemodynamics of the cavernous sinus, causing venous vasculitis and further causing cluster-like headache. Alternatively, a tumor can cause direct compression of the nerve located in the brain stem, affecting the brain stem autonomic reflex. In addition, carotid bodies, which are sensitive to hypoxia stimulation, may be involved. As in these previous cases, our patient was also male, with a history of meningioma, a long headache-onset period, and insensitivity to conventional treatment, all of which are suggestive of non-primary cluster headache. However, unlike these other cases, our patient experienced the onset of cluster-like headache 2.5 years after surgical removal of the meningioma. Therefore, we infer that the pathogenesis of our patient was different from and more complicated than that in the previous cases. It remains unclear whether his headaches were due to recurrence of the tumor, long-term complications of the surgery, or postoperative glial hyperplasia in the frontal-temporal lobe.

**Table 1:** Cluster-like headache secondary to anamnesis of sphenoid ridge meningioma: a review of the literature.

**Case and reference number**	**1(11)**	**2(18)**	**3(19)**	**4(20)**	**5(21)**
Headache onset age (years)	29	30	<40	23	68
Sex	Male	Male	Male	Male	Male
Disease duration (years)	8	–	>20	23	0.5
Cluster-like headache form	Episodic	Chronic	Chronic	Chronic	–
Location of meningioma	Left planum sphenoidale	Left cerebellopontine angle	Right posterior fossa	Left-sided parasellar	The right side of the lower medulla and upper cervical cord
Pathological type /grade	Atypical meningioma/grade II	Choroid meningioma/grade II	Transitional meningioma/-	–	–
Response to routine treatment	–	Partially resolved with verapamil and sumatriptan	Prednisone and verapamil reduced the intensity	Only steroids had a significant effect	No treatment was effective
Outcome of meningioma resection	Complete amelioration	Complete amelioration	Complete amelioration	Complete amelioration	Complete amelioration
Possible mechanisms	Ipsilateral cavernous sinus may be involved	–	Autonomic reflexes in the brain stem may be involved	Orbital venous vasculitis and cavernous sinus may be involved	Autonomic outflow in the brain stem and carotid body activated by hypoxemia may be involved

The brain MRI indicated local dural thickening and enhancement; however, no significant increase of the enhancement volume was observed during follow-up. The ^18^F-FDG PET scan also showed no signs of a tumor. However, we cannot completely rule out the possibility of tumor recurrence. On one hand, for tumors such as meningioma, which grow very slowly, a significant increase in the volume on the MRI may not be observed in a 6-month period. On the other hand, although ^18^F-FDG is currently the most commonly used PET tracer in oncology, it may not reliably detect chronically growing tumors such as meningioma. In some cases, meningioma has been reported to show as much ^18^F-FDG uptake as normal gray matter, while others were characterized with hypometabolic features, resulting in a low ratio of tumor-to-gray matter uptake (22–24). Even in the same transitional meningioma, ^18^F-FDG PET may reveal different imaging characteristics according to histopathologic differences (25). Thus, the cluster-like headache may have been caused by recurrence of meningioma, located in the region of the sphenoid ridge near the trigeminal nerve, which may have activated the left trigeminovascular system and parasympathetic nerve fibers (26).

Acute and chronic pain frequently occurs after craniotomy. The common prevalent patterns are tension-type and migraine-like headaches, while cluster-like headache is rare. A report described four patients presenting with delayed-onset temporal pain after pterional craniotomy, ipsilateral to the operative site, radiating around the eye, and lasting from 10 min to 1 h (27). Significant orofacial pain, temporomandibular dysfunction, and trigeminal sensitization on the ipsilateral side of the pterional craniotomy is reported (28). These reports suggest that craniotomy may damage nearby nerves, including the trigeminal nerve, which may have been a risk factor for cluster-like headache in our patient.

In our patient, postoperative changes in the left frontal-temporal lobe were significant. A large area of softening lesion and glial hyperplasia was visible on a brain MRI scan performed in March 2018 (Figures 3A,B). Previous studies have found that neuronal-glial communication is involved in the development of neuronal sensitization and transduction of painful stimuli within the trigeminal ganglion, which are likely to play an important role in the pathogenesis of migraine (29, 30). Therefore, we speculate that the hyperplasic glial cells in the left frontal-temporal lobe may have secreted excessive inflammatory factors, stimulating local nerves and blood vessels, which activated the trigeminal nervous system and caused the cluster-like headache.

Last but not least, by reviewing the literature, we found that a new MRI scan sequence, three dimensional-fast imaging employing steady state acquisition (3D-FIESTA), which has extremely high spatial resolution, can clearly distinguish nerves, and blood vessels with low signal and cerebrospinal fluid with high signal, thus plays an important role in the diagnosis of intracranial neurovascular contact compression (31, 32). Therefore, MRI with 3D-FIESTA protocol will be recommended on the patient's return, and that any findings from the imaging will be further communicated.

Here, we reported the rare case of a patient with anamnesis of a sphenoid ridge meningioma, and the original location of the meningioma was consistent with that of a subsequently developed cluster-like headache. Occult recurrence of meningioma, pterional craniotomy, and glial cell hyperplasia may have caused the cluster-like headache via stimulation of the left trigeminal nerve and vascular system alone or in combination.

## Ethics Statement

The case report was conducted in accordance with the ethical standards set out in the Helsinki Declaration of 1964. Written informed consents were obtained from the patient for the participation and publication of this case report.

## Author Contributions

All the authors were involved in patient management and made patient treatment decisions. TW, LK, and JiH conceived the idea, drafted the manuscript, and revised all the literature. YaX, CH, and KM collected the clinical data. XG, FW, and SY analyzed and interpreted the brain images. YuX and GZ revised the Discussion by adding important points. LL and NX participated in the revision of the manuscript. The manuscript has been read and approved by all the named authors.

### Conflict of Interest Statement

The authors declare that the research was conducted in the absence of any commercial or financial relationships that could be construed as a potential conflict of interest.
